# Pencil Lead as a Material for Microfluidic 3D-Electrode Assemblies

**DOI:** 10.3390/s18114037

**Published:** 2018-11-19

**Authors:** Emilia Witkowska Nery, Magdalena Kundys-Siedlecka, Yoshitaka Furuya, Martin Jönsson-Niedziółka

**Affiliations:** Institute of Physical Chemistry, Polish Academy of Sciences, Kasprzaka 44/52, 01-224 Warsaw, Poland; mkundys@ichf.edu.pl (M.K.-S.); yfuruya@iis.u-tokyo.ac.jp (Y.F.)

**Keywords:** pencil graphite electrode (PGE), microfluidics, 3D electrode

## Abstract

We present an electrochemical, microfluidic system with a working electrode based on an ordered 3D array of pencil leads. The electrode array was integrated into a plexiglass/PDMS channel. We tested the setup using a simple redox probe and compared the results with computer simulations. As a proof of concept application of the device we showed that the setup can be used for determination of dopamine concentration in physiological pH and ultrasensitive, although only qualitative, detection of *p*-nitrophenol with a limit of detection below 1 nmol L^−1^. The observed limit of detection for *p*-nitrophenol is not only much lower than achieved with similar methods but also sufficient for evaluation of exposure to pesticides such as methyl parathion through urinalysis. This low cost setup can be fabricated without the need for clean room facilities and in the future, due to the ordered structure of the electrode could be used to better understand the process of electroanalysis and electrode functionalization. To the best of our knowledge it is the first application of pencil leads as 3D electrochemical sensor in a microfluidic channel.

## 1. Introduction

Pencil leads consist of high quality graphite powder (27–60%) mixed with mica, to which a polymeric binder is sometimes added [1]. As graphite is one of the most popular electrode materials (together with precious metals such as Au and Pt) it is only reasonable that researchers are trying to take advantage of this easily available and low-cost electrode substrate. When compared with other electrode materials pencil graphite is extremely low cost, and thus can be disposable. It is readily available all over the world, does not require polishing and in some cases presents higher sensitivity than glassy carbon electrodes (a characteristic attributed to a higher surface area) [2,3]. Graphite’s sp^2^ hybridization results in its high conductivity as well as strong adsorption properties [3].

The first reports concerning the use of pencil graphite as an electrode material date back to 1954 when pencil served as a reference electrode for polarographic measurements (cited in [4]). Afterwards commercial pencils found application as: anodes for polarography [4], electrodes and diffracting objects in hydrodynamic spectroelectrochemical studies [5] and finally as working electrodes for stripping voltammetry [6]. Nowadays pencil graphite electrodes (PGE) can be used directly, or embedded in insulating polymers and micropipette tips. Graphite rods are also used to draw electrodes on paper in order to achieve fully disposable and low cost paper-based analytical devices [7].

**Figure 1:**
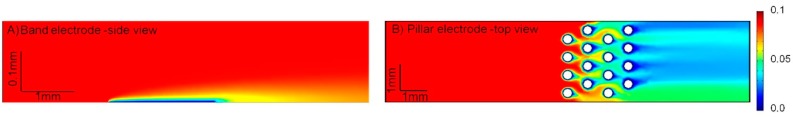
Concentration profiles of the analyte (mol L^−1^) in a simplified electrochemical reaction in a microfluidic channel calculated using COMSOL. (**A**) using a microband electrode (side view) and (**B**) using 16 vertical posts as electrodes (top view). The channel dimensions, flow rates (*V_f_*) and over-potential are the same in both systems. Red color denotes high concentration and blue color low concentration. In (A) the collection efficiency is a mere 3% compared to 81.5% in (B).

**Figure 2:**
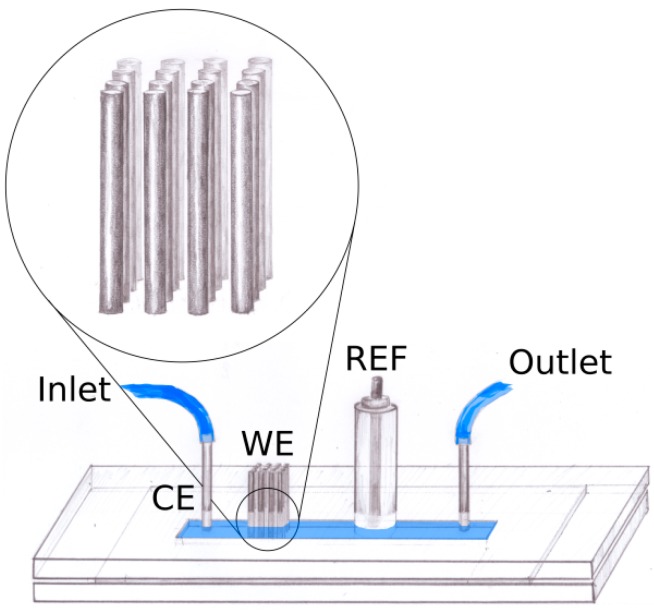
Schematic drawing of the device, CE—counter electrode, WE—working electrode, RE—reference electrode.

We would like to exploit one more asset of pencils, which, as far as we are aware, has as yet gone unmentioned, i.e., their format. The diameter of novel pencil leads can be as small as 100 µm in case of test devices [8] and 200 µm for commercially available graphite rods (e.g., the nominal diameter of a Pentel Orenz lead is 0.2 mm, but in reality they are 0.25 mm). This size allows the fabrication of 3D assemblies of graphite posts which can be enclosed in microfluidic channels forming chemical and biochemical reactors or sensors. Two main advantages of this approach are: (1) more efficient utilization of the analyte; depending on the size of the electrode and the flow rate as little as few percent of the analyte might be detected in a typical experiment using traditional microband electrodes [9,10,11], sample calculations are presented in Figure 1; (2) the regular structure of the proposed electrode array is easier to simulate than a non-ordered 3D assembly (e.g., in the form of a sponge) which can help to better understand both functionalization of the electrode material and electroanalysis using such assemblies. Until now three-dimensional electrodes were mainly explored in dielectrophoretic separation [12], or as parts of capacitors [13] and fuel cells [14] but not so much in sensing.

Pencil graphite electrodes are also rarely applied in microfluidic systems. Pencils can be arranged along the side walls of the microchannel forming microelectrodes for studies on electrokinetic phenomena (alternating current electroosmosis, induced-charge electro-osmosis, and dielectrophoresis) [15] or embedded in the bottom of the channel to form microband electrodes used for hydroxyl radical detection [16]. The three dimensionality of graphite rods was, as far as we are aware, only explored in a microfluidic fuel cell in which pencil leads were stacked along the channel. That system consisted of 12 pencil anodes, 12 cathodes and five spacers filling the channel to its entire height. This assembly allowed for single pass fuel utilization levels up to 78% [17].

Several groups have drawn attention to differences between pencils of different grade, which determines the clay/graphite ratio, and from different manufactures, which both should be taken into account when working with this kind of electrodes. Detailed information regarding composition and electrochemical behavior of different pencil leads can be found in the literature [18,19].

In this paper we describe the construction and characterization of a microfluidic device equipped with an array of 16 pencil leads combined into a working electrode. The detection of *p*-nitrophenol and dopamine, the latter in the presence of the interfering substances ascorbic and uric acids, were chosen as a proof of concept applications of the proposed device.

*p*-Nitrophenol is one of the simplest phenol derivatives and it is also listed as a priority pollutant by the U. S. Environmental Protection Agency [20]. Applications of *p*-nitrophenol include pesticides, fungicides, organic dyes and pharmaceuticals [2]. It causes formation of methemoglobin, liver and kidney damage and can lead to death at high exposure levels [20,21]. *p*-Nitrophenol persists in industrial and agricultural wastewaters, not only as a direct contaminant but also as a biodegradation product of parathion and methyl parathion [21]. Urinary *p*-nitrophenol can serve as a biomarker of exposure to the abovementioned pesticides [22].

Dopamine is one of the most important neurotransmitters—crucial for the brain’s reward and motor systems. Like other catecholamines, dopamine is also biosynthesized from a tyrosine precursor. Also as most catecholamine neurotransmitters, dopamine is electroactive and can be detected on a number of electrode materials, including metals and carbon [23,24]. The biggest challenge in the detection of this compound in physiological pH are the interfering species, such as ascorbic acid, which concentration can be as high as 0.5 mmol L^−1^ in the extracellular fluid of the brain [24].

## 2. Materials and Methods

### 2.1. Device Fabrication

The pencil leads were positioned in a 4 × 4 grid of orifices which were drilled in two pieces of 1.5 mm poly(methyl methacrylate) (PMMA) using a computer controlled micromilling machine. Both layers of PMMA also contained four holes for the standard screws used for assembly of the device and two orifices used for positioning. Inlet and outlet as well as an orifice for the reference electrode of standard construction were drilled in one of the layers. The microfluidic channel (0.3 × 3 × 20 mm) was fabricated in a polydimethylsiloxane (PDMS) gasket using a 3D printed nylon mold. The gasket was positioned between the two PMMA parts. The device is schematically presented in Figure 2. To achieve proper alignment of the electrodes, pencil leads were pushed through the orifices in the top and bottom PMMA layers. In initial experiments only one positioning layer was used, but electrochemical studies showed very low reproducibility of such systems, as it was not possible to guarantee that all electrodes passed through the whole height of the channel all the way to the bottom.

### 2.2. Electrochemical Characterization

The electrochemical cell consisted of a three-dimensional pencil graphite electrode (4 to 16 leads), an Ag/AgCl (sat. KCl) reference electrode of standard construction and a steel counter electrode which also formed the inlet of the device. All leads (Pentel Orenz HB 0.2 mm) were connected to form one working electrode but in principle each lead could also be addressed separately. All electrochemical experiments were performed on a PGSTAT 20 potentiostat (Metrohm Autolab, Herisau, Switzerland). A syringe pump (Pump 11 Elite, Harvard Apparatus, Holliston, MA, USA) was used to ensure constant flow rate. In order to characterize the device a series of experiments with 1 mmol L^−1^ ferrocenedimethanol (FcDM) in 0.1 mol L^−1^ KNO_3_ was carried out. The flow rate was varied from 0 to 1000 µL min^−1^ and the electrode consisted of 4 to 16 graphite posts (from 1 to 4 lines of 4 pencil leads). Measurements were performed on at least three devices for each of the four electrode configurations.

Significant drift of the signal was observed for the first measurements in each new device, thus an electrochemical pretreatment procedure was implemented. The potential was scanned from −0.1 to 0.5 V, at 100 mV s^−1^ shifting the flow rate from 25 to 750 µL min^−1^ until the signal reached a stable value (usually around 75 scans). The need for electrochemical activation may arise from the porous nature of the pencil graphite electrode. It was already shown that electrochemical pretreatment of such electrodes increases their active surface area as well as their hydrophilicity by formation of oxygenated functionalities (i.a. phenolic, carbonyl, carboxyl). Such pretreatment also ensures the cleanliness of the electrode surface [2,3].

### 2.3. Computer Modeling Using COMSOL Multiphysics

Computations were performed with COMSOL Multiphysics 5.2a on a computer equipped with an IntelCore i7-3930 processor, 48 GB of RAM and a Windows operating system. A single calculation was usually completed within 2–8 min. The calculated electrochemical response of the system was based on a two-dimensional model of the microfluidic channel with pencil pillars (see Figure 1B). First, the hydrodynamic conditions inside the channel were calculated with application of the Laminar Flow (spf) module. The calculated flow was used in the Transport of Diluted Species (tds) module to determine the electrochemical response, which was described using Butler-Volmer-type equations. The simulation of the electrochemical reaction $Red⇄Ox+ne^{-}$ was made with application of the fluxes of both oxidized and reduced species at the electrode surface. The inward flux at the electrode surfaces is described by the equations:

(1) $$\Phi_{0,Ox}=k_{f}\left[Ox\right]-k_{b}\left[Red\right]$$

(2) $$\Phi_{0,Red}=-k_{b}\left[Ox\right]+k_{f}\left[Red\right]$$

The forward *k_f_*, and reverse *k_b_*, rate constants were represented by:

(3) $$k_{f}=k_{0}\exp\left(-\alpha \frac{nF}{RT}\eta \right)$$

(4) $$k_{b}=k_{0}\exp\left(\left(1-\alpha \right)\frac{nF}{RT}\eta \right)$$

where *k*_0_ is the standard rate constant, *Ox* and *Red* were used as the concentration of the oxidized and reduced form of the redox species, respectively, *η* represents the over-potential, *α* is the transfer coefficient, *n* number of electrons, *F* the Faraday constant, *R* the universal gas constant, and *T* the temperature. The current was calculated by integration of $\Phi_{0,Ox}nF$ over the surface of the pencil pillars. For the calculation we used *k*_0_ = 5 × 10^−6^ m s^−1^, D = 1× 10^−9^ m^2^ s^−1^, *α* = 0.5, *n* = 1. In the case of resistive posts, the applied potential at the electrode was calculated as $\eta_{R}=\eta -RI$, where *R* is the resistance and *I* the calculated current. $\eta_{R}$ was then used instead of η in Equations (3) and (4).

### 2.4. Neurotransmitter Sensing

First tests were performed using pencil leads prepared with:

(1) the standard pretreatment procedure –cycling between −0.1 to 0.5 V in 1 mmol L^−1^ FcDM, 0.1 mol L^−1^ KNO_3_,
(2) oxidized by cycling between 1.2 to 1.8 V in 0.1 mol L^−1^ NaOH solution, 25 cycles, 100 mV s^−1^
(3) oxidized by application of constant potential of 1.8 V in PBS solution (pH 7.4) for 600 s, the latter was shown to result in a graphene like surface when applied to glassy carbon electrodes [25]. In this case pre-treatment was applied to enhance the electrode performance (we evaluated the impact on the current and peak separation while performing measurement of the interferents).

After analysis of the SEM images and voltammetric data ‘graphene’ pretreatment was chosen for consecutive tests. Cyclic voltammograms registered in a solution of FcDM after application of abovementioned pretreatment procedures are presented in Figure S5 of the Supplementary Information (SI).

Both square wave, and differential pulse voltammetry were used to perform initial tests but SWV resulted in better resolved peaks in case of mixtures and higher signal to noise ratio. The measurement parameters were as follows: frequency 8 Hz, step 0.00105 V, amplitude 50 mV, scan rate 84 mV s^−1^. Measurements were performed in physiological pH (7.4) to mimic the environment of real samples.

### 2.5. p-Nitrophenol Assay

Preliminary studies confirmed strong adsorption of *p*-nitrophenol on graphite, which resulted in a significant decrease of the oxidation peak for each consecutive scan. Therefore it was necessary to find a regeneration protocol, which could be applied after each measurement to clean the electrode surface from the adsorbed *p*-nitrophenol. A literature search was performed in order to select potential cleaning protocols, which could be applied to regenerate the electrode surface in a microfluidic setup, as standard treatment based on mechanical removal of the deposit through polishing could not be applied in our system. The tested regeneration protocols included:

(1) electrochemical oxidation in 0.2 mol L^−1^ NaCl, pH 3, different potentials (up to 4.5 V) and times of treatment (up to 20 min) tested;
(2) electrochemical oxidation in 1.5% H_2_O_2_, 0.2 mol L^−1^ NaCl, pH 3 [26], different potentials (up to 4.5 V) and times of treatment (up to 20 min) tested;
(3) adsorptive displacement using iodine and thiosulfate [27]. After each measurement solution of 40 mmol L^−1^ of iodine in ethanol was passed for 10 min (25 µL min^−1^), next electrochemical cell was cleaned with deionized water. Chemical reduction of iodine to iodide was achieved using 0.5 mol L^−1^ sodium thiosulfate in water (3 min, 25 µL min^−1^), after which the system was cleaned with distilled water and considered ready for the next measurement.
(4) NaOH oxidative cycling which was earlier applied as a pretreatment step in the dopamine assay. After each measurement, the surface was regenerated with 25 scans between 1.2 to 1.8 V in 0.1 mol L^−1^ NaOH solution.

Adsorptive displacement (method no. 3) and NaOH oxidative cycling (method no. 4) were chosen for subsequent studies as oxidation in hydrogen peroxide allowed for the regeneration of the electrode surface only at very high potentials (4.5 V) and intense evolution of gas accompanying the procedure, resulted in leakage of the microfluidic system, when the procedure was applied repeatedly. Up to 20 min treatment in sodium chloride did not exert any positive effect on the electrode surface.

After optimization of the pH, measurement parameters and the regeneration protocol, electrochemical analysis was performed in pH 4.0 acetate buffer in case of adsorptive displacement protocol (3) and in PBS pH 7.4 in case of NaOH oxidative cycling (4) using Differential Pulse Voltammetry from 0.2 to 1.25 V, with a 5 mV step, 150 mV modulation amplitude and 50 ms modulation time, scan rate 10 mV s^−1^.

## 3. Results

### 3.1. Characterization

**Figure 3:**
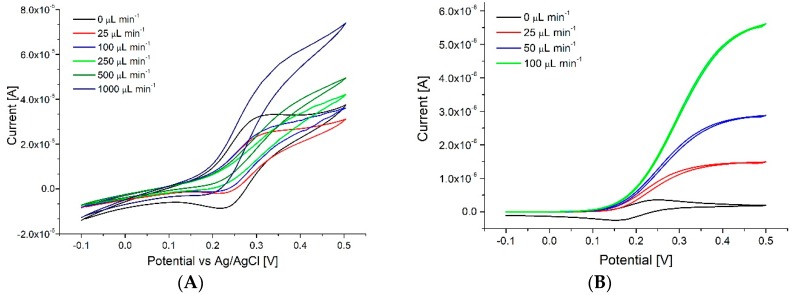
(**A**) Cyclic voltammetry studies of oxidation of FcDM performed for different flow rates (0–1000 µL min^−1^). Electrode cell consisted of a 3D working electrode formed from 16 graphite posts, steel counter electrode and an Ag/AgCl reference. Scan rate 100 mV s^−1^. (**B**) Calculated cyclic voltammograms for the 16 electrode post system.

**Figure 4:**
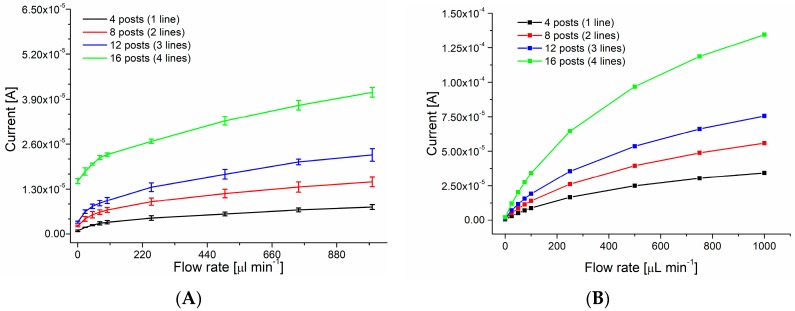
Limiting current registered for 3D working electrodes formed from four to 16 graphite posts (four posts/one line –black, eight posts/two lines –red, 12 posts/three lines –blue, 16 posts/four lines –green). Chronoamperometry performed at 0.35 V vs. Ag/AgCl. (**A**) Experimental data, standard deviation calculated for n = 3 devices, (**B**) Calculated data.

As shown in Figure 3A the shift between diffusion (peak shaped) to convection (sigmoidal shaped voltammogram) controlled mass transport [28] occurs around 100 µL min^−1^, which is slightly higher than observed for calculations (50 µL min^−1^, Figure 3B). The difference can be partially attributed to the fact that calculations were performed in 2D, thus they do not take into account slower flow observed near the top and bottom of the channel. Chronoamperometry measurements presented expected results with steady increase of the limiting current from four, eight to 12 posts. In the experimental data a higher increase was observed between 12 to 16 posts (three to four lines of graphite rods) which may be due to disturbances in the flow caused by the more complex grid of electrodes. Despite the complex structure of the system, the reproducibility between devices, as seen in Figure 4A was quite high (mean value of standard deviation around 3% of the signal for assemblies of 16 pencil posts).

The results of the simulations show (Supporting Information Figure S2), that the current increases almost linearly with increasing flow rate. At first sight this is in disagreement with the experimental results. However, the chronoamperometry was measured at low overpotential (at 0.35 V vs. Ag/AgCl/KCl_sat_), and the system possesses some uncompensated resistance as well. Adding these parameters to the simulations reproduces the experimental data quite well (Figure 4B).

The almost linear increase of the current with *V_f_* might be surprising if compared to the behavior of a microband electrode, where the limiting current scales as *V_f_*^1/3^. A somewhat better comparison might instead be the wall-jet, where the power is ¾ so a power close to unity does not seem unreasonable. Although mass transport to a cylinder in uniform flow is a classic problem in fluid dynamics (see e.g., [29]), with approximate analytical solutions, we could not find any analyses concerning cylinders in a channel. Calculations of flow and heat transfer in fin-tube heat exchangers are very similar to our system, but usually at much higher Reynolds numbers [30]. At low flow rates, when the Peclet number between the pillars is below 40 we see an increased influence of diffusion (indicated by a Sherwood number as low as 2–3). We also see that the depletion zones between the pillars start overlapping (cf. zone III and IVa in [9]). This leads to a decrease in the current. Results of those calculations are shown in Supporting Information Figures S3 and S4. We can see this more clearly in the experimental data since the 2D simulation does not take into account the slower flow close to the top and bottom of the channel which exacerbates the effect of low flow rate.

### 3.2. SEM Analysis

**Figure 5:**
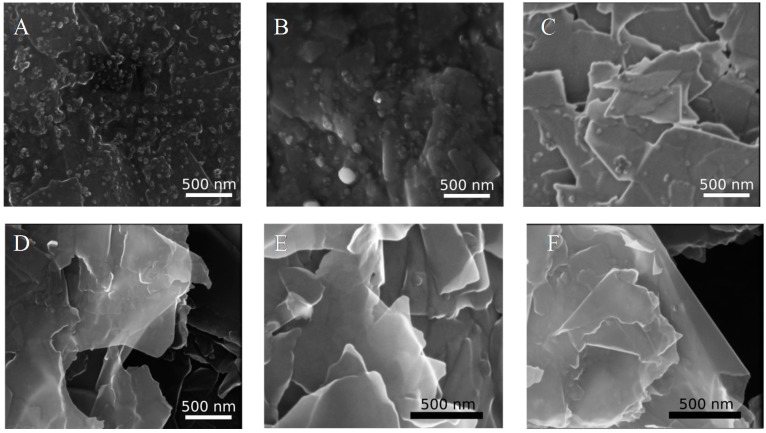
SEM images of pencil electrodes (side of the pencil shaft) magnification 50,000×: (**A**) before modification, (**B**) after cycling between −0.1 to 0.5 V (**C**) after oxidation in NaOH, (**D**) after “graphene” treatment. Magnification 75,000×: (**E**) after oxidation in NaOH, (**F**) after “graphene” treatment. Analysis performed with a Nova NanoSEM 450 microscope.

Figure 5 shows SEM images of pencil electrodes before and after electrochemical pretreatment. After oxidation, individual flakes of graphite can be discerned, resulting in a much higher surface area as compared to the compact structures of mildly treated and untreated pencils. EDX analysis indicated that the granules seen on untreated pencil lead are mainly formed from Zn, and are mostly removed even by mild electrochemical treatment (Figure 5A,B).

### 3.3. Neurotransmitter Detection

Both oxidative pretreatment procedures resulted in about one order of magnitude increase in the current in case of dopamine sensing, compared to pencils cycled only in mild conditions. Comparison of the current increase after different procedures is presented in SI Figure S5. This can probably be attributed to the enhanced surface area also noted in the SEM images. Prolonged cycling in NaOH (Figure 5C,E) resulted in a less reproducible signal, which might be related to loosely attached thin graphite flakes, which would detach upon touch and at higher flow rates (particles are seen in the outflow during longer experiments). This negative effect was not observed on pencils oxidized at constant potential in PBS (‘graphene’ pretreatment Figure 5D,F). Therefore this pretreatment was chosen for subsequent experiments.

**Figure 6:**
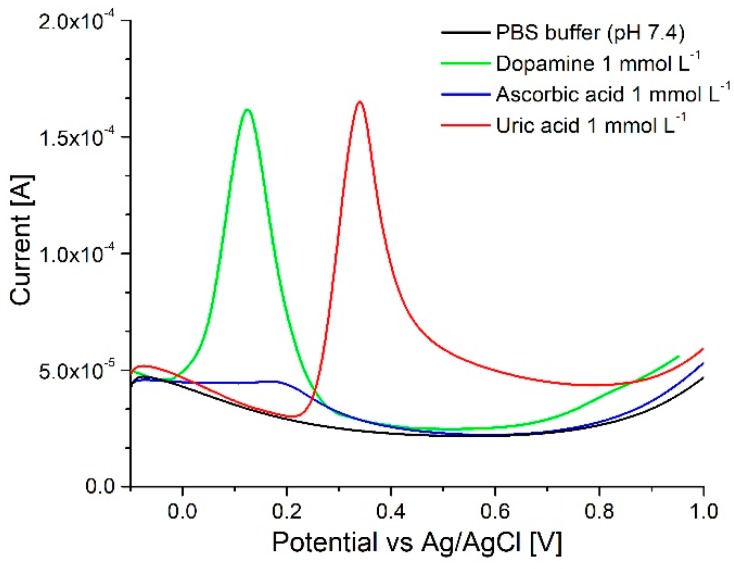
Square wave voltammetry of equimolar solutions of dopamine (green), uric acid (red) and ascorbic acid (blue) recorded using the array device. Array of 16 posts, flow of 100 µL min^−1^, pencils oxidized at constant potential in PBS.

**Figure 7:**
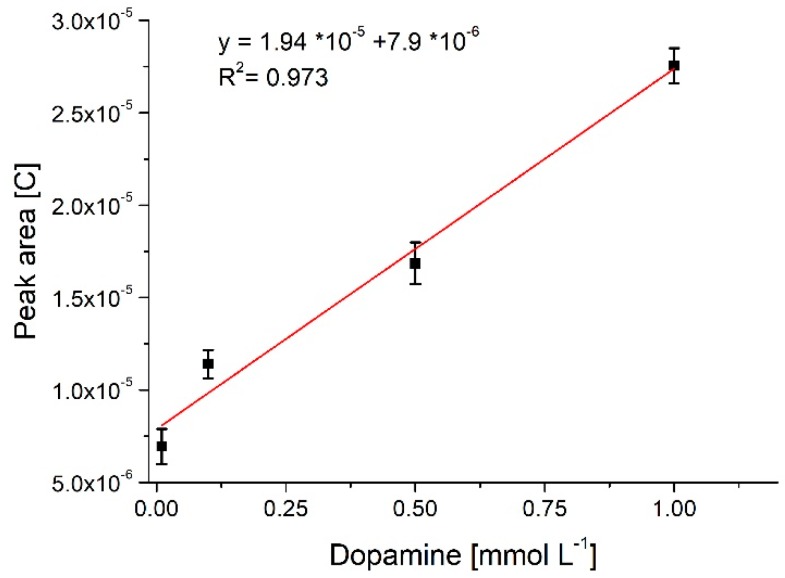
Calibration curve for dopamine, standard deviation calculated for n = 3. Array of 16 posts, flow of 100 µL min^−1^, pencils oxidized at constant potential in PBS.

As seen in Figure 6 it was possible to differentiate dopamine and uric acid using the oxidized ‘graphene’ like pencil leads. Even though separation of ascorbic acid was not completely possible, the registered signal for this compound is sufficiently low as compared with uric acid and dopamine to allow reliable analysis in most sample types. Tests were performed with dopamine concentrations ranging from 100 nmol L^−1^ to 10 mmol L^−1^ and as can be seen in Figure 7 a close to linear response was registered for samples between 10 µmol L^−1^ to 1 mmol L^−1^ after which saturation occurred. Sample square wave voltammograms used for the construction of the calibration curve as presented in SI Figure S6. These measurements were performed at physiological pH 7.4. It is known that dopamine can polymerize to block the electrode at this high pH [11] but no such negative effect was observed in the presented system.

### 3.4. p-Nitrophenol Assay

#### 3.4.1. Adsorptive Displacement (Method n° 3)

**Figure 8:**
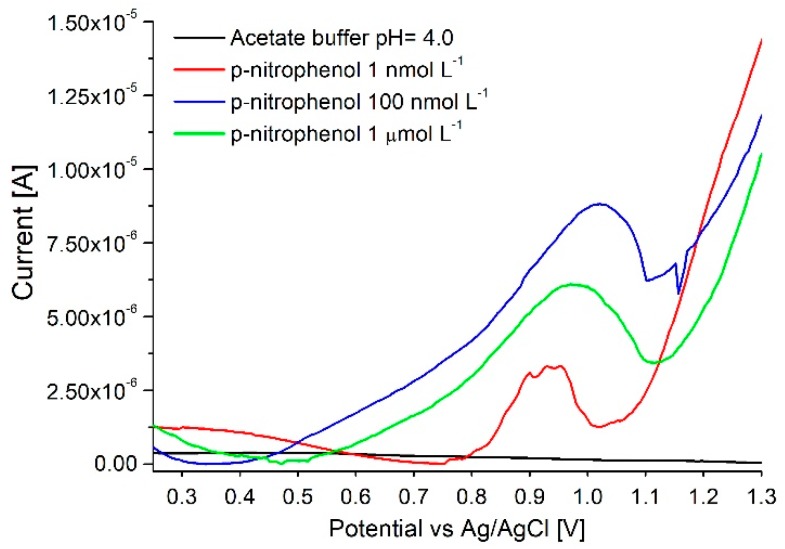
Detection of p-nitrophenol on pencil electrodes. Array of 16 posts, flow of 100 µL min^−1^, pencils regenerated using adsorptive displacement, with iodine.

Although it was possible to clearly discern samples containing as low as 1 nmol L^−1^ of *p*-nitrophenol from blank buffer samples the analysis was only qualitative (Figure 8). Although the chosen regeneration protocol allowed to fully clean the electrode from adsorbed *p*-nitrophenol (stable measurements in buffer solution, detection of p-nitrophenol possible after each regeneration) as seen in Figure 8 the current was not reproducible - the signal for 1 µmol L^−1^ can be lower than for 100 nmol L^−1^. This behavior may be attributed to changes in the electrode surface after the regeneration. Changes in flow rate, the time of the iodine displacement or recovery steps did not greatly influence the reproducibility of the measurement.

#### 3.4.2. NaOH Oxidative Cycling (Method n° 4)

**Figure 9:**
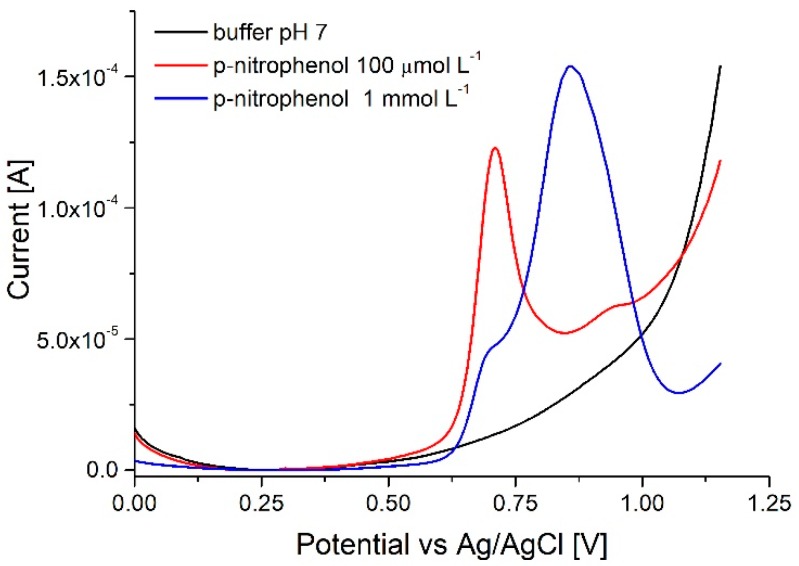
Two-step oxidation of p-nitrophenol on electrodes regenerated using NaOH oxidative cycling (the same method as tested for dopamine detection). Array of 16 posts, flow of 100 µL min^−1^.

The NaOH regeneration protocol used subsequently allowed for more consistent results in terms of baseline and the level of the signal; unfortunately it induced other problems. After treatment with NaOH the *p*-nitrophenol peak split in two. It was already described by Jiang et al. [31] that in alkaline solutions oxidation of nitrophenols is mostly a two-step process with two peaks corresponding to oxidation of a phenolate to phenoxy radical and subsequently to phenoxy cation. Regardless the time a buffer solution was passed through the channel after NaOH treatment, the electrode reaction of *p*-nitrophenol was still a two-step process (Figure 9) where the ratio between the peaks was inconsistent between experiments. Inconsistencies in peak ratio can be attributed to different retention of the base solution between graphite flakes and difficulties associated with measuring of such two-step reactions in flow conditions.

Although our assay only provided qualitative information, the response for concentrations as low as 1 nmol L^−1^ was still easily distinguishable from the background, which is well below limits of detection provided by other pencil lead devices described for this compound (1.1 µmol L^−1^ for the paper-based assay [21], 7.48 µmol L^−1^ for bismuth-modified pencil [32], 1.9 µmol L^−1^ for copper modified pencil electrodes [33] and 11 nmol L^−1^ for a multiwalled carbon nanotube/graphite pencil electrode in a flow injection system with a pre-concentration step [34]). The ability of the system to clearly discern samples with such low concentration (1 nmol L^−1^) is enough to clearly discern people suffering from pesticide poisoning (e.g., methyl parathion) in need of relocation and treatment from “no action needed” groups, including more strict norms applicable to children from 1 to 16 years of age [22].

This system can be fabricated even in resource limited settings, without the need for clean room facilities, and uses only readily available materials and reagents thus it could potentially serve as a screening tool for pesticide poisoning in parts of the globe where microfabrication techniques are still a luxury. This kind of system could potentially be used together with smartphone-based detectors [35] and in this way reach a wider public.

## 4. Discussion

We have presented a novel microfluidic device equipped with a 3D electrode assembly of regular structure which can be fabricated without the use of clean room facilities. Even though experiments were performed using a commercial reference electrode, setup was also tested with a homemade reference, constructed according to [36] proving that all three electrodes could be fabricated from readily available materials (syringe needle, pencil leads, glass capillary). Experimental characterization of the device was backed with computer simulations which demonstrates proper functioning of the system and let us believe that this kind of device can serve in the future to better explain mechanisms behind electroanalytical assays as well as elaborate methods of electrode functionalization. Our system is extremely versatile as the proposed fabrication method allows for development of different electrode setups and the pencil rods can be replaced with other electrode materials such as gold or platinum wires.

To further characterize our device we chose two proof-of-concept applications: dopamine and *p*-nitrophenol sensing. We have shown the possibility of dopamine detection in the range from 10 µmol L^−1^ to 1 mmol L^−1^, which is comparable with other works on pencils [37,38] but is still high when set together with many currently described electrodes, or the physiological range (basal concentration of dopamine ~20 nmol L^−1^ in rat cerebrospinal fluid [39]). Detection of dopamine in the presence of interferents, in a physiological range is a separate challenge, as it is often impossible to separate peaks of different compounds when measured in mixture [40]. To achieve this separation electrodes are often modified with different catalysts, which was already presented by us [11] and many others. Quite limited blocking of the electrode, even at physiological pH observed in our system is a clear advantage. The detection limit and linear range could be improved with appropriate electrode modification which we plan to explore in the future, taking advantage of the architecture of our device. To address the problem of measurement of complex mixtures, or real samples, electrodes in each line of the device could be modified with different materials, thus providing the possibility of machine learning assisted discrimination (electronic tongue).

The second proof-of-concept application, the detection of *p*-nitrophenol, proved to be especially troublesome. This compound was already detected using pencil graphite electrodes, but the paper-based assay with drawn electrodes [21] took advantage of the low price of pencils and disposability of such devices. When not enclosed in a microfluidic channel the electrode surface could also easily be regenerated by polishing off the layers with adsorbed analyte. In our case each consecutive assay had to be preceded with a regeneration step, and even though several procedures were tested restoration of the initial electrode surface proved to be a challenge (lack of contamination after regeneration but notable changes in surface area). Nevertheless the response for concentrations as low as 1 nmol L^−1^ was still easily distinguishable from the background. Such a low level of detection allows to assess pesticide poisoning through urinalysis. Therefore a system of this kind—easy to fabricate and operate (thanks to enclosure of the electrodes in a microfluidic channel), using readily available materials and methods of fabrication, could potentially serve as a point of care device in resource limiting settings. 

## Supplementary Materials

The following are available online at http://www.mdpi.com/1424-8220/18/11/4037/s1, Figure S1: Cyclic voltammetry studies of oxidation of FcDM performed for different flow rates, Figure S2: Limiting current calculated for 3D working electrodes, Figure S3: Calculated concentration profiles along a cut-line, Figure S4: Calculated 2D concentration profiles, Figure S5: Cyclic voltammetry of FcDM after different pretreatments, Figure S6: Square wave voltammetry of dopamine solutions of different concentrations.

## References

[1] Bhowmik R.N. (2012). Ferromagnetism in lead graphite-pencils and magnetic composite with CoFe_2_O_4_ particles. Compos. Part B.

[2] Kawde A., Baig N., Sajid M. (2016). Graphite pencil electrodes as electrochemical sensors for environmental analysis: A review of features, developments, and applications. RSC Adv..

[3] David I.G., Popa D., Buleandra M. (2017). Pencil Graphite Electrodes: A Versatile Tool in Electroanalysis. J. Anal. Methods Chem..

[4] Mašek J. (1960). A simple microcoulometric arrangement for polarographic purposes using the three-electrode system. J. Electroanal. Chem..

[5] Demana T., Peterman D., Shaffer J., Melaragno P.R. (1989). Flow-Through Spectroelectrochemical Detector Based on Diffraction at a Cylindrical Electrode. Anal. Chem..

[6] Blum D., Leyffer W., Holze R. (1996). Pencil-Leads as new electrodes for abrasive stripping voltammetry. Electroanalysis.

[7] WitkowskaNery E., Santhiago M., Kubota L.T. (2016). Flow in a Paper-based Bioactive Channel—Study on Electrochemical Detection of Glucose and Uric Acid. Electroanalysis.

[8] Sharp Announcement “0.1” Which Is Not Broken Even with Ultra-fine 0.2 Is also Reference Exhibited. www.nichima.co.jp/news/entry/1747.html.

[9] Amatore C., Da Mota N., Sella C., Thouin L. (2007). Theory and experiments of transport at channel microband electrodes under laminar flows. 1. Steady-state regimes at a single electrode. Anal. Chem..

[10] Amatore C., Da Mota N., Sella C., Thouin L. (2010). Theory and experiments of transport at channel microband electrodes under laminar flow. 3. Electrochemical detection at electrode arrays under steady state. Anal. Chem..

[11] Rozniecka E., Jonsson-Niedziolka M., Celebanska A., Niedziolka-Jonsson J., Opallo M. (2014). Selective electrochemical detection of dopamine in a microfluidic channel on carbon nanoparticulate electrodes. Analyst.

[12] Martinez-Duarte R. (2012). Microfabrication technologies in dielectrophoresis applications—A review. Electrophoresis.

[13] Jiang H., Lee S., Li C. (2013). 3D carbon based nanostructures for advanced supercapacitors. Energy Environ. Sci..

[14] La O’ G.J., In H.J., Crumlin E., Barbastathis G., Shao-Horn Y. (2007). Recent advances in microdevices for electrochemical energy conversion and storage. Int. J. Energy Res..

[15] Bashirzadeh Y., Maruthamuthu V., Qian S. (2016). Electrokinetic Phenomena in Pencil Lead-based Microfluidics. Micromachines.

[16] Ouyang J., Li Z.-Q., Zhang J., Wang C., Wang J., Xia X.-H., Zhou G.-J. (2014). A rapid and sensitive method for hydroxyl radical detection on a micro fl uidic chip using an N-doped porous carbon nano fiber modified pencil graphite electrode. Analyst.

[17] Kjeang E., McKechnie J., Sinton D., Djilali N. (2007). Planar and three-dimensional microfluidic fuel cell architectures based on graphite rod electrodes. J. Power Sources.

[18] Navratil R., Kotzianova A., Halouzka V., Opletal T., Triskova I., Trnkova L., Hrbac J. (2016). Polymer lead pencil graphite as electrode material: Voltammetric, XPS and Raman study. J. Electroanal. Chem..

[19] Kariuki J.K. (2012). An Electrochemical and Spectroscopic Characterization of Pencil Graphite Electrodes. J. Electrochem. Soc..

[20] Wu Z., Yuan X., Zhong H., Wang H., Zeng G., Chen X., Wang H., Zhang L., Shao J. (2016). Enhanced adsorptive removal of p-nitrophenol from water by aluminum metal–organic framework/reduced graphene oxide composite. Sci. Rep..

[21] Santhiago M., Henry C.S., Kubota L.T. (2014). Low cost, simple three dimensional electrochemical paper-based analytical device for determination of p-nitrophenol. Electrochim. Acta.

[22] Hryhorczuk D.O., Moomey M., Burton A., Runkle K., Chen E., Saxer T., Slightom J., Dimos J., Mccann K., Barr D. (2002). Urinary p -Nitrophenol As a Biomarker of Household Exposure to Methyl Parathion. Environ. Health Perspect..

[23] Perry M., Li Q., Kennedy R.T. (2009). Review of recent advances in analytical techniques for the determination of neurotransmitters. Anal. Chim. Acta.

[24] Robinson D.L., Hermans A., Seipel A.T., Wightman R.M. (2008). Monitoring rapid chemical communication in the brain. Chem. Rev..

[25] Santhiago M., Maroneze C.M., Silva C.C.C., Camargo M.N.L., Kubota L.T. (2015). Electrochemical Oxidation of Glassy Carbon Provides Similar Electrochemical Response as Graphene Oxide Prepared by Tour or Hummers Routes. ChemElectroChem.

[26] Zhou M.H., Lei L.C. (2006). Electrochemical regeneration of activated carbon loaded with p -nitrophenol in a fluidized electrochemical reactor. Electrochim. Acta.

[27] Manes M. (1977). Recovery of Activated Carbon. U.S. Patent.

[28] Bard A.J., Faulkner L.R. (2001). Potential sweep methods. Electrochemical Methods Fundamentals and Applications.

[29] Friedlander S.K. (1957). Mass and heat transfer to single spheres and cylinders at low Reynolds numbers. AIChE J..

[30] Wung T.-S., Chen C.J. (1989). Finite Analytic Solution of Convective Heat Transfer for Tube Arrays in Crossflow: Part II—Heat Transfer Analysis. J. Heat Transf..

[31] Jiang Y., Zhu X., Li H., Ni J. (2010). Effect of nitro substituent on electrochemical oxidation of phenols at boron-doped diamond anodes. Chemosphere.

[32] Asadpour-Zeynali K., Najafi-Marandi P. (2011). Bismuth modified disposable pencil-lead electrode for simultaneous determination of 2-nitrophenol and 4-nitrophenol by net analyte signal standard addition method. Electroanalysis.

[33] Kawde A.N., Aziz M.A. (2014). Porous Copper-Modified Graphite Pencil Electrode for the Amperometric Detection of 4-Nitrophenol. Electroanalysis.

[34] Gholivand M.B., Khodadadian M., Bahrami G. (2015). Molecularly Imprinted Polymer Preconcentration and Flow Injection Amperometric Determination of 4-Nitrophenol in Water. Anal. Lett..

[35] Wo A., Hogan C., Elton D., Loke S. (2017). Mobile voltammetric analysis. International Patent.

[36] East G.A., del Valle M.A. (2000). Easy-to-Make Ag/AgCl Reference Electrode. J. Chem. Educ..

[37] Galván-Valencia M., Albino-Navarro G., Flores-Morales V., Durón-Torres S.M. (2008). Pencil Electrodes for Determination of Dopamine, Serotonine and Ascorbic Acid. ECS Trans..

[38] Özcan A., Yücel S. (2009). Selective and sensitive voltammetric determination of dopamine in blood by electrochemically treated pencil graphite electrodes. Electroanalysis.

[39] Shou M., Ferrario C.R., Schultz K.N., Robinson T.E., Kennedy R.T. (2006). Monitoring dopamine in vivo by microdialysis sampling and on-line ce-laser-induced fluorescence. Anal. Chem..

[40] Tukimin N., Abdullah J., Sulaiman Y. (2018). Review—Electrochemical Detection of Uric Acid, Dopamine and Ascorbic Acid. J. Electrochem. Soc..

