# Supplementary material for: Pencil Lead as a Material for Microfluidic 3D-Electrode Assemblies
Source: Sensors (Basel). 2018 Nov 19;18(11):4037. doi: 10.3390/s18114037 (PMC6264025; doi:10.3390/s18114037)
Supplement: Supplementary file 1 [file sensors-18-04037-s001.pdf]

Supporting Information

Pencil lead as a material for microfluidic 3D-electrode assemblies

Emilia Witkowska Nery *, Magdalena Kundys-Siedlecka, Yoshitaka Furuya and Martin Jönsson-Niedziółka *

Institute of Physical Chemistry, Polish Academy of Sciences, Kasprzaka 44/52, 01-224 Warsaw, Poland; mkundys@ichf.edu.pl (M.K.-S.); yfuruya@iis.u-tokyo.ac.jp (Y.F.)

* Correspondence: ewitkowskanery@ichf.edu.pl (E.W.N.); martinj@ichf.edu.pl (M.J.-N.); Tel.: +48-22-343-3306 (E.W.N. & M.J.-N.)

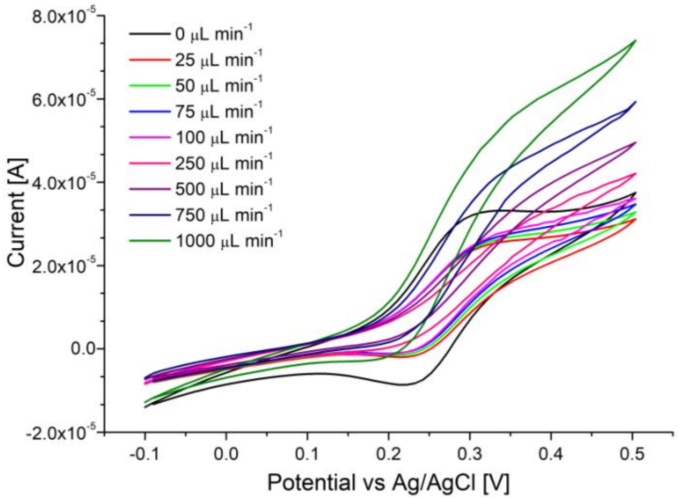

SI Figure 1. Cyclic voltammetry studies of oxidation of FcDM performed for different flow rates (0-1000 μL min^{-1}). Electrode cell consisted of a 3D working electrode formed from 16 graphite posts, steel counter electrode and an Ag/AgCl reference. Scan rate 100 mV s^{-1}.

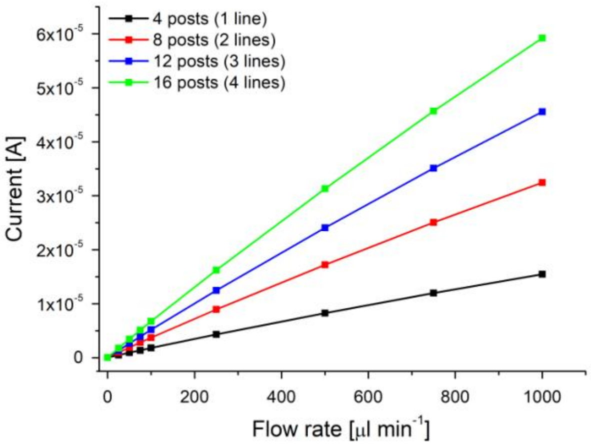

SI Figure 2. Limiting current calculated for 3D working electrodes formed from 4 to 16 graphite posts.

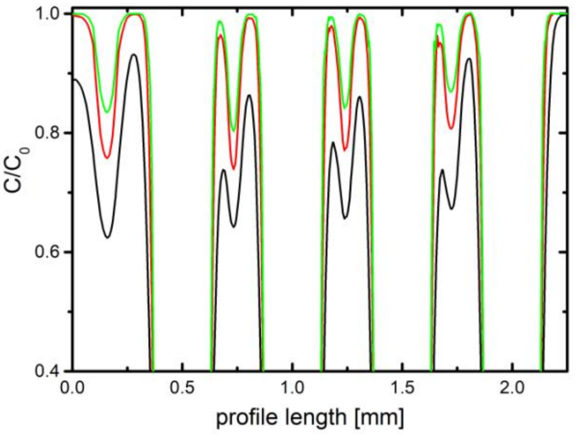

SI Figure 3. Concentration profile of the reduced form of the redox probe along a cut-line through the second row of electrodes for different flow rates: black 10 µl min^{-1}, red 25 µl min^{-1}, green 100 µl min^{-1}. The profile is taken at the turning point in the CV (fig. 3b) at 0.6 V.

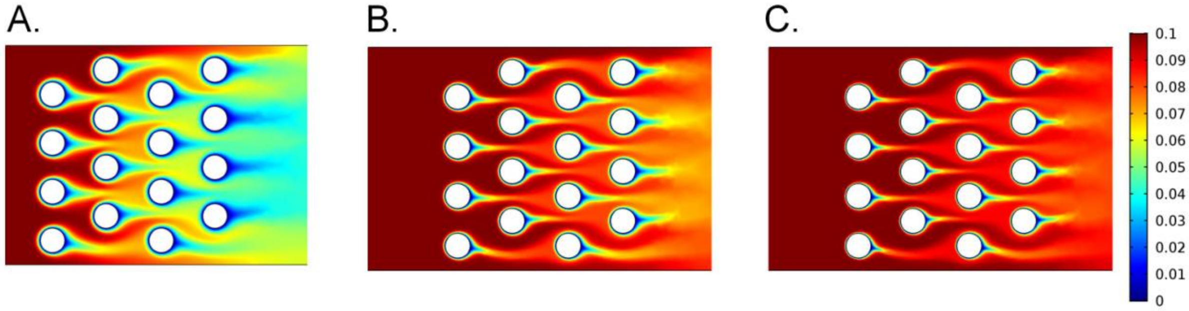

SI Figure 4. Concentration profile of the reduced form of the Redox probe around the electrodes for different flowrates: A) 10 µl min^{-1}, B) 25 µl min^{-1}, C) 100 µl min^{-1}. The image is saved at the turning point in the CV (fig. 3B) at 0.6 V. The flow is from the left to the right side.

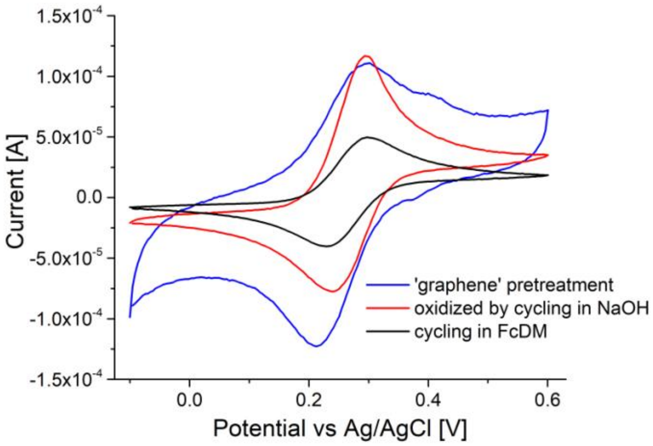

SI Figure 5. Cyclic voltammetry of FcDM performed using a pencil graphite electrode: prepared by cycling in FcDM, oxidized by cycling in NaOH and oxidized by applying constant potential in PBS.

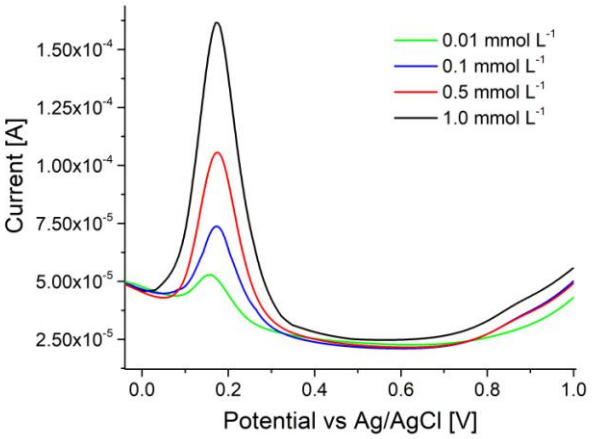

SI Figure 6. Square wave voltammetry of dopamine solutions recorded using the array device, corresponding to the calibration curve presented in the manuscript in Fig. 7
